# Silencing C_19_-GA 2-oxidases induces parthenocarpic development and inhibits lateral branching in tomato plants

**DOI:** 10.1093/jxb/erv300

**Published:** 2015-06-19

**Authors:** Liliam Martínez-Bello, Thomas Moritz, Isabel López-Díaz

**Affiliations:** ^1^Instituto de Biología Molecular y Celular de Plantas, Universidad Politécnica de Valencia (UPV)-Consejo Superior de Investigaciones Científicas (CSIC), Ingeniero Fausto Elio s/n, 46022 Valencia, Spain; ^2^Umeå Plant Science Centre, Department of Forest Genetics and Plant Physiology, Swedish University of Agricultural Science, S-90183 Umeå, Sweden

**Keywords:** Branching, GA 2-oxidases, gibberellins, parthenocarpy, silencing, tomato.

## Abstract

Gibberellins (GAs) are phytohormones that regulate a wide range of developmental processes in plants. Levels of active GAs are regulated by biosynthetic and catabolic enzymes like the GA 2-oxidases (GA2oxs). In tomato (*Solanum lycopersicum* L.) C_19_ GA2oxs are encoded by a small multigenic family of five members with some degree of redundancy. In order to investigate their roles in tomato, the silencing of all five genes in transgenic plants was induced. A significant increase in active GA_4_ content was found in the ovaries of transgenic plants. In addition, the transgenic unfertilized ovaries were much bigger than wild-type ovaries (about 30 times) and a certain proportion (5–37%) were able to develop parthenocarpically. Among the *GA2ox* family, genes *GA2ox1* and *-2* seem to be the most relevant for this phenotype since their expression was induced in unfertilized ovaries and repressed in developing fruits, inversely correlating with ovary growth. Interestingly, transgenic lines exhibited a significant inhibition of branching and a higher content of active GA_4_ in axillary buds. This phenotype was reverted, in transgenic plants, by the application of paclobutrazol, a GA biosynthesis inhibitor, suggesting a role for GAs as repressors of branching. In summary, this work demonstrates that GA 2-oxidases regulate gibberellin levels in ovaries and axillary buds of tomato plants and their silencing is responsible for parthenocarpic fruit growth and branching inhibition.

## Highlight

GA 2-oxidases regulate gibberellin levels in ovaries and axillary buds of tomato plants and their silencing is responsible for parthenocarpic fruit growth and branching inhibition.

## Introduction

**Fig. 1.**
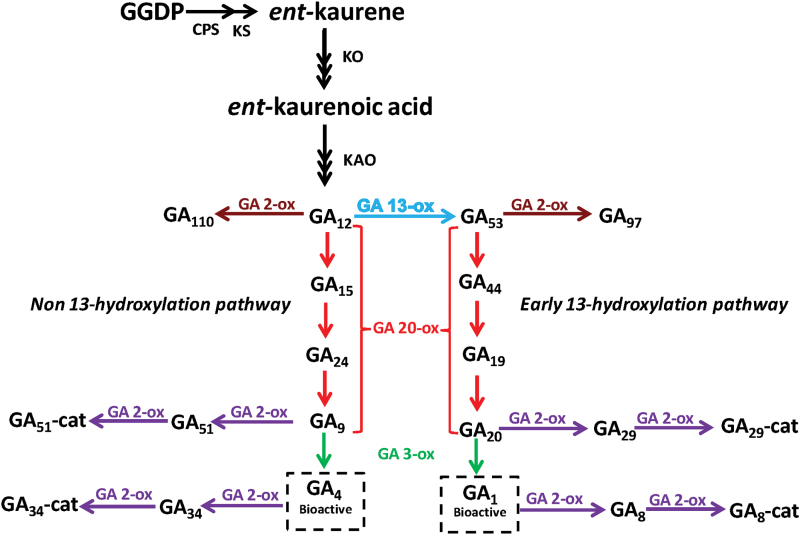
GA biosynthesis pathway. GGDP, geranylgeranyl diphosphate; CPS, ent-copalyl diphosphate synthase; KS, ent-kaurene synthase; KO, ent-kaurene oxidase; KAO, ent-kaurenoic acid oxidase; GA13ox, GA 13-oxidase; GA20ox, GA 20-oxidase; GA3ox, GA 3-oxidase; GA2ox, GA 2-oxidase; GA-cat, GA-catabolite. (This figure is available in colour at *JXB* online.)

Gibberellins (GAs) are phytohormones that regulate a wide range of developmental processes in plants such as germination, stem elongation, leaf expansion, flowering, fruit-set, and fruit growth (Yamaguchi, 2008; Mutasa-Göttgens and Hedden, 2009; Seymour *et al.*, 2013). GA biosynthesis (Fig. 1) starts with geranyl geranyl diphosphate (GGDP) which is converted to *ent*-kaurene by the action of two enzymes: CPS (*ent*-copalyl diphosphate synthase), and KS (*ent*-kaurene synthase). The *ent*-kaurene is then converted to GA_12_, the first GA of the pathway, by the action of two membrane-associated P450 enzymes (cytochrome P450 mono-oxygenases): *ent*-kaurene oxidase (KO) and *ent*-kaurenoic acid oxidase (KAO). From this step, there are two parallel pathways: the non-13-hydroxylation (leading to active GA_4_) and the early 13-hydroxylation (leading to active GA_1_), which depend on the action of the cytochrome P450 enzyme, GA 13-oxidase (GA13ox) (Magome *et al.*, 2013). The enzymes that catalyse the last steps of the biosynthesis pathways are GA 20-oxidases (GA20ox) and GA 3-oxidases (GA3ox) which are 2-oxoglutarate dependent dioxygenases (2ODDs) (Yamaguchi, 2008; Hedden and Thomas, 2012).

The GA metabolic pathway is tightly controlled in order to keep adequate hormone levels which are necessary for the fine regulation of plant growth and development. Endogenous GA levels are regulated, mainly, by biosynthesis and inactivation. Several mechanisms for inactivating GAs have been identified, the most prevalent being 2β-hydroxylation. The enzymes responsible for this activity are GA 2-oxidases (GA2oxs) which are also 2ODDs, like the GA20ox and GA3ox enzymes (Thomas *et al.*, 1999; Rieu *et al.*, 2008; Hedden and Thomas, 2012). The GA2ox enzymes can be divided into two major groups according to substrate specificity: one group acts on the GAs with 19 carbons (C_19_-GA2oxs), which include the bioactive GAs, GA_1_ and GA_4_, and their immediate precursors GA_20_ and GA_9_ (Serrani *et al.*, 2007a; Hedden and Thomas, 2012), whereas the other group acts on GAs with 20 carbons (C_20_-GA2oxs) GA_12_ and GA_53_ (Schomburg *et al.*, 2003). Tomato (*Solanum lycopersicum* L.) is one of the most important crops worldwide. It has been widely used, not only as a food but also as a model plant due to several developmental traits not found in other model plants such as *Arabidopsis thaliana, Antirrhinum majus*, and rice (*Oryza sativa* L.). These traits are sympodial shoot branching, compound leaves, and the formation of fleshy climacteric fruits (Ranjan *et al.*, 2012). In addition, tomato has several characteristics that make it a convenient model plant species, such as a relatively compact genome (950Mb) combined with a marker-saturated genetic linkage map, rich germplasm collections (Tomato Genetics Resource Center), and highly efficient transformation protocols (Carvalho *et al.*, 2011). The Micro-Tom (MT) cultivar has been proposed as a very convenient tomato model system because of its small size, rapid life cycle, and high-throughput capabilities (Meissner *et al.*, 1997, Carvalho *et al.*, 2011). The phenotype of this cultivar is the result of point mutations in the genes Dwarf (D), Self-pruning (SP), and Internode length reduction (Ilr) but not in GA biosynthesis (Martí *et al.*, 2006). Serrani *et al* (2007b) demonstrated that this cultivar is an appropriate model for research on the hormonal regulation of fruit set and development because it responds to GAs and auxins in the same way as other tomato cultivars. The isolation and characterization of GA genes (Serrani *et al*, 2007a), GA mutants (Carrera *et al*, 2012), and GA transgenic plants (Garcia-Hurtado *et al.*, 2012) in this cultivar, is another argument in favour of its choice for the study of developmental processes mediated by GA.

In tomato, there are five GA2ox genes that encode for enzymes that belong to the group of C_19_-GA2oxs. Sequence analysis of amino acid composition showed that *GA2ox2*, *-4*, and *-5* belong to subgroup I and *GA2ox1* and *-3* belong to subgroup II (Serrani *et al.*, 2007a). The *GA2ox* genes are redundantly expressed in vegetative and reproductive tissues of tomato. In order to understand the roles of *GA2ox* genes in tomato development, transgenic plants were generated in which the expression of all *GA2ox* genes was silenced. The transgenic plants showed the induction of facultative parthenocarpy and a significant inhibition in the appearance of lateral branches and also a significant increase in the GA_4_ concentration in ovaries and axillary buds compared with wild type (WT) plants. This suggests that *GA2ox* genes are important for the regulation of GA levels in the ovaries and axillary buds, thus playing an important role in regulating their development.

## Materials and methods

### Accession numbers

Accession numbers for the sequences used in this study are as follows: *GA2ox1* (EF441351), *GA2ox2* (EF441352), *GA2ox3* (EF441353) *GA2ox4* (EF441354), *GA2ox5* (EF441355), and *SlActin* (U60482.1).

### Plant material and growth conditions

Tomato (*S. lycopersicum* L.) cv. Micro-Tom (MT) was used. Plants were put in a growth chamber at 26±2 °C under 16/8h light/dark (photon fluence of 115 μmol m^–2^ s^–1^) conditions. In the greenhouse, plants (one per pot) were grown in 1 L pots with a mixture of peat:vermiculite (1:1 v/v) in 24/20 ºC day/night conditions and irrigated daily with Hoagland’s nutrient solution (Hoagland and Arnon, 1950). Natural light was supplemented with Osram lamps (Powerstar HQI-BT, 400W) to get a 16h light photoperiod.

### Generation of constructs for silencing

A short-hairpin RNA (shRNA) construct was used to induce multiple silencing of all the *GA2ox* genes. To ensure its efficiency, a chimeric fragment was used composed of portions identical to the *GA2ox1, GA2ox3*, and *GA2ox4* genes and very similar to *GA2ox2* and *GA2ox5* (more than 92% identity). This fragment was composed of a portion of 163bp from *GA2ox4*, 188bp from *GA2ox1*, and 123bp from *GA2ox3* (see Supplementary Table S1 at *JXB* online). Each fragment was amplified separately from cDNA clones from Serrani *et al.* (2007a) using primers described in Supplementary Table S2 at *JXB* online. These fragments were purified and joined by an overlapping PCR as shown in Supplementary Fig. S1 at *JXB* online. The final fragment shRNA2ox was cloned in the pGEM-T Easy vector (Promega) and sequenced to confirm that the sequences were correct and had been well assembled. To create the final construct for tomato plants transformation, Gateway technology was used using the primers described in Supplementary Table S2 at *JXB* online. The fragment was recombined into the binary vector pK7GWIWG2 (II) (Karimi *et al*., 2002), using LR Clonase (Invitrogen) to obtain the construct *35S::GA2ox*/RNAi. This vector carries the constitutive promoter 35S from *Cauliflower mosaic virus* (CaMV) and the *nptII* gene for kanamycin resistance.

### Isolation of transgenic tomato plants

Construct *35S::GA2ox*/RNAi was used for transformation using the *Agrobacterium tumefaciens* strain LBA4404. Tomato transformation was carried out as described in García-Hurtado *et al.* (2012).

### Germination conditions

Tomato seeds from transgenic lines and WT plants were imbibed in 90mm diameter plastic Petri dishes on three layers of Whatman No. 1 filter paper (Fisher) with double deionized water (ddH2O). Petri dishes were sealed with Parafilm (Fisher) to prevent evaporation and put in a growth chamber, as previously described. Germination was scored when the radicle protruded from the seed coat.

### Hypocotyl and root length measurements in seedlings

Seeds from each WT and transgenic line were germinated (no more than 20 seeds per jar) and cultured in germination medium (GM) [Murashige and Skoog (MS) salts, 1% (w/v) sucrose, and 0.8% (w/v) agar] in a growth chamber. After 7 d, the seedlings were photographed and the hypocotyls and roots were measured using the ImageJ software (National Institutes of Health, USA).

### Branching measurements

The branching pattern of transgenic *35S::GA2ox/RNAi* and WT plants was determined by measuring the percentage of branched axils, calculated as: % Branching=(no. of branched axils/total no. of axils)×100. Branched axils are those axils with a branch equal or longer than 0.5cm.

### Hormone application in plants

For this experiment, 27-d-old plants grown in pots in the greenhouse were used. Paclobutrazol (PCB) and gibberellic acid (GA_3_) from Duchefa were first dissolved in absolute ethanol and then diluted with the nutrient solution used to water the plants. The GA_3_ concentration used was 10^–5^ M and the PCB concentration was 10^–6^ M. Plants were treated on alternate days for 16 d, a total of eight applications. As a control group, plants were watered with nutrient solution containing the same amount of absolute ethanol used to dissolve PCB and GA_3_. For each line a total of 15 plants per treatment were used.

### Determination of parthenocarpic capacity

To determine parthenocarpic capacity, three flowers per truss were selected from the first four trusses of the plant. Flower emasculation was carried out 2 d before anthesis to prevent self-pollination and all non-selected flowers from the truss were removed. Only those fruits bigger than 0.1g, 20 d after anthesis, were considered to be parthenocarpic.

### RNA isolation

Total RNA was isolated from reproductive and vegetative tissues, using the RNeasy Plant Mini Kit (Qiagen) according to the manufacturer’s protocol. Total RNA was treated with RNase-Free DNAse (Qiagen), according to the manufacturer’s instructions. RNA concentration was measured using a Nanodrop ND-1000 Spectrophotometer (Wilmington, Delaware USA) and RNA integrity was determined by agarose gel analysis. Three biological replicates for each sample were used. For the ovaries, only those from the first four inflorescences were used and only three ovaries per inflorescence.

### Quantitative Real-Time RT-PCR analysis (RT-qPCR)

One microgram of total RNA was used for cDNA synthesis using the High Capacity cDNA Reverse Transcription Kit (Applied Biosystems) with random hexamers. Aliquots (2 μl) of diluted cDNA solution (1:2 v/v) were used for qPCR reactions in a final volume of 20 μl. PCR reactions were performed in an optical 96-well plate on a 7500 Fast Real-Time PCR System using Power SYBR Green PCR Master Mix (Applied Biosystems). The analyses were carried out using three biological replicates and three technical replicates. The primer pairs used for PCR amplification were the same as in Serrani *et al.* (2008) (see Supplementary Table S3 at *JXB* online). For each pair of primers the optimal concentration and efficiency in the PCR reaction was determined. In every run a template-free control was included. To be able to compare data from different runs, the C_T_ value of every gene was normalized with the C_T_ value of *SlActin* determined in the same run. Relative expression of mRNA was calculated by the 2^–ΔΔCT^ method (Livak and Schmittgen, 2001).

### GA quantification

GAs were quantified in reproductive and vegetative tissues. Apical shoots, stems, and axillary buds were chosen as the vegetative tissues. Apical shoots (apices and the three youngest leaves) were collected after flowering but before anthesis, when the biggest flower in the first inflorescence was no more than 4mm. For this analysis the flowers were removed. Seven biological replicates of five plants each were used for WT plants and four biological replicates of five plants each for transgenic line L1. Stems were taken from 27-d-old plants from the first to the fifth internode over the cotyledons, once the apical shoot and the leaves were removed. Three biological replicates of three plants each were used. Axillary buds, which did not show any visible growth at the time of harvest, were taken from the 3rd, 4th, and 5th axil of the plant and three biological replicates of 15 plants each were used. For reproductive tissue, 10-d-old hand-pollinated ovaries were chosen and five biological replicates of four ovaries each were used. The material was frozen immediately in liquid N_2_ before storage at –70 °C until analysis.

The extraction procedure for apical shoots and ovaries was performed as follows. Aliquots of 100mg fresh weight were homogenized in cold 80 % (v/v) methanol-H_2_O, stirred overnight at 4 °C and re-extracted twice for 30min with one-half volume of methanol. The methanolic extracts were combined and taken to dryness in a vacuum and dissolved in 0.5ml of 10% methanol solution. This extract was then applied to a pre-equilibrated 500mg SAX column (BondElut SS-SAX; Varian Scharlau). The column was washed with H_2_O pH 8.0 before being eluted with 4ml of 0.2M formic acid solution. The formic-acid eluate was run directly onto a pre-equilibrated 500mg C_18_ column (BondElut C_18_; Varian Scharlau) which was then washed with H_2_O pH 3.0 and finally eluted with 5ml 80% methanol. The methanol eluate was dried, dissolved in 0.2ml of 2-propanol and then methylated with trimethylsilyl-diazomethane in hexane. After methylation, the residue was dried and the samples were trimethylsilylated using 7.5 µl of pyridine and 7.5 µl of BSTFA solution containing 1% TCMS (N,O-*bis*(trimethylsilyl) trifluoroacetamide+1% trimethyl chlorosilane). For gas chromatography–mass spectrometry analysis (GC–MS), the samples were dissolved in the mix of pyridine/BSTFA and injected into a gas chromatograph (7890A; Agilent Technologies) coupled to a mass spectrometer (7000 Triple Quad; Agilent Technologies). The amount of GA in the samples was quantified by isotopic dilution, using the MassHunter Quantitative software provided by Agilent.

The extraction procedure for GA quantification in stems and axillary buds was performed as follows. Aliquots (about 100–200mg fresh weight) of frozen material were extracted with 80% methanol–1% acetic acid, and the extracts passed consecutively through HLB (reverse phase), MCX (cationic exchange), and WAX (ionic exchange) columns (Oasis 30mg, Waters), as described in Seo *et al.* (2011). The final residue was dissolved in 5% acetonitrile–1% acetic acid, and the hormones were separated using an autosampler and reverse phase UPHL chromatography (2.6 μm Accucore RP-MS column, 50mm length×2.1mm i.d.; ThermoFisher Scientific) with a 5–50% acetonitrile gradient containing 0.05% acetic acid, at 400 μl min^–1^. The hormones were analysed by electrospray ionization (negative mode, spray voltage 3.0kV, heater temperature 150 ºC, sheath gas flow rate 40 μl min^–1^, auxiliary gas flow rate 10 μl min^–1^) and targeted-SIM using a Q-Exactive spectrometer (Orbitrap detector; ThermoFisher Scientific). The concentrations of hormones in the extracts were determined using embedded calibration curves and the Xcalibur 2.2 SP1 build 48 and TraceFinder programs.

The deuterium-labelled hormones (purchased from Professor L Mander, Canberra, Australia, OlChemim Ltd, Olomouc, Czech Republic, and the Cambridge Isotope Laboratory, Andover, USA) were added to the extracts as internal standards for quantification of each of the different GAs. In the case of axillary buds and stems, GA_34_ was not available and could not be quantified.

### Statistical methods

Statistical treatments of the data were made using the SPSS program, version 16.0 for windows, IBM. The analyses were made by t-student and one-way ANOVA for p<0.05. As post-hoc tests we use Bonferroni and Tukey’ HSD.

## Results

### Isolation of transgenic tomato lines

The five *GA2ox* genes of tomato are redundantly expressed according to Serrani *et al.* (2007a) suggesting functional redundancy which had previously been demonstrated in *Arabidopsis* (Rieu *et al.*, 2008). In order to study the role of *GA2ox* genes, the whole tomato *GA2ox* family was silenced. As described in the Material and methods, tomato leaf explants were infected with *Agrobacterium tumefaciens* LBA4404 carrying the *35S::GA2ox/RNAi* short-hairpin construct. Six independent diploid lines were obtained: L1, L5, L13, L22, L25, and L81. These were grown in pots and cultured in the greenhouse. Their progenies, obtained by self-pollination, were tested for kanamycin resistance to determine the segregation of the transgene, but only three of them had a 3:1 proportion, corresponding to a single locus of the transgene (L1, L5, and L81). Homozygous lines from these plants, carrying the *35S::GA2ox/*RNAi transgene, were isolated and lines L1 and L5 were chosen for extensive characterization.

### Silencing of *GA2ox* genes in transgenic plants

**Fig. 2.**
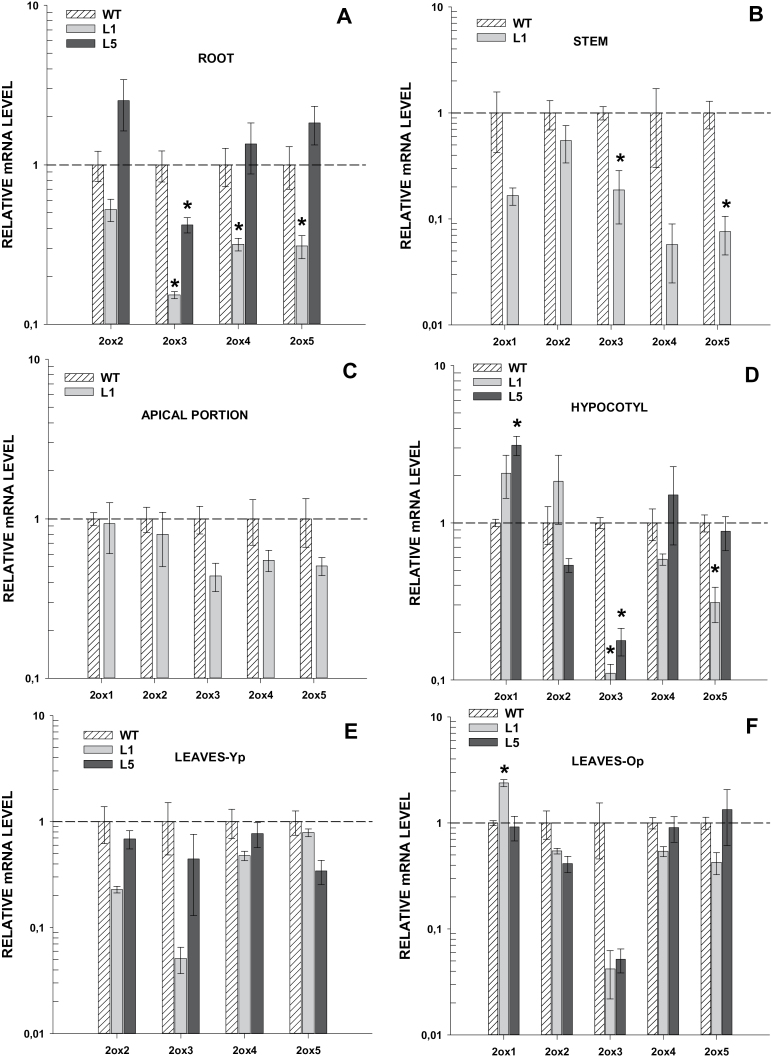
Silencing of *GA2ox1–5* in vegetative tissues of transgenic *35S::GA2ox/RNAi* plants. Expression of *GA2ox* genes in vegetative tissues of wild-type (WT) plants and transgenic lines L1 and L5. (A) Roots (from 90-d-old plants), (B) stems (27-d-old plants, approximately 1 week before anthesis), (C) apical shoots (apical portions consisting of shoot apices and three apical young leaves before anthesis), (D) hypocotyls (7-d-old seedlings), (E) leaves-Yp (terminal leaf from young flowering plants), and (F) leaves-Op (leaves from the apical portion of 90-d-old plants). Values are means of three biological replicates ±SE and are relative to the value of expression in the WT plants (1.0). *, Significantly different from the WT plants (*P* <0.05).

**Fig. 3.**
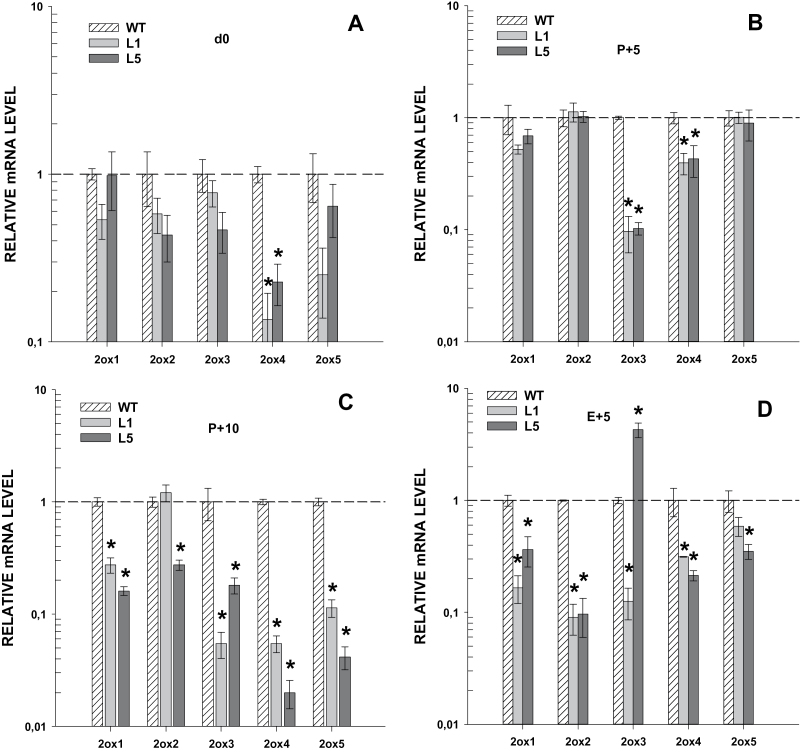
Silencing of *GA2ox1–5* in reproductive tissues of transgenic *35S::GA2ox/RNAi* plants. Expression of *GA2ox* genes in reproductive tissues of wild-type (WT) plants and transgenic lines L1 and L5. (A) d0 (ovaries at the time of anthesis), (B) P+5 (pollinated ovaries 5 d post-anthesis), (C) P+10 (pollinated ovaries 10 d post-anthesis) and (D) E+5 (emasculated ovaries 5 d post-anthesis). Values are means of three biological replicates ±SE and are relative to the value of expression in the WT plants (1.0). *, Significantly different from the WT plants (*P* <0.05).

**Fig. 4.**
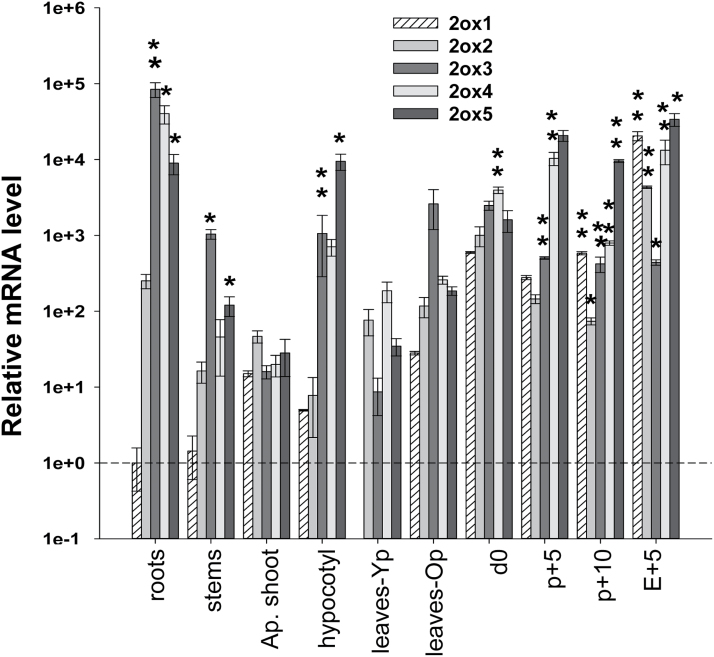
Relation between abundance of mRNA levels of *GA2ox* genes and silencing in vegetative and reproductive tissues of transgenic plants. Bars represent the relative abundance of *GA2ox* genes in different tissues and those labelled with asterisks are those genes silenced in at least one transgenic line. The *SlActin* gene was used as an internal control for each tissue and values are means of three biological replicates ±SE. All data are expressed relative to the values of *GA2ox1* expression in roots, set as 1.0. **, The gene is silenced in two transgenic lines; *, the gene is silenced in only one transgenic line. The vegetative tissues were taken from Roots (from 90-d-old plants), Stems (27-d-old plants, approximately 1 week before anthesis), Apical shoots (apical portions consisting of shoot apices and three apical young leaves before anthesis), Hypocotyls (from 7-d-old seedlings), Leaves-Yp (apical young leaves of 35-d-old plants), and Leaves-Op (apical young leaves of 90-d-old plants). The reproductive tissues were taken from ovaries: d0 (ovaries at the time of anthesis), P+5 (pollinated ovaries 5 d post-anthesis), P+10 (pollinated ovaries 10 d post-anthesis), and E+5 (emasculated ovaries 5 d post-anthesis).

To determine the efficiency of silencing of transgene *35S::GA2ox/RNAi*, the expression of the *GA2ox* genes was analysed in vegetative and reproductive tissues. In vegetative tissues, significant silencing of genes *GA2ox3, GA2ox4*, and *GA2ox5* was detected in roots and of *GA2ox3* and *GA2ox5* in hypocotyls and stems (Fig. 2). Genes *GA2ox1* and *GA2ox2* were not silenced in any of the vegetative tissues analysed. In reproductive tissues, by contrast, silencing of all the *GA2ox* genes was detected (Fig. 3). These results indicate that the *35S::GA2ox/RNAi* construct is able to induce silencing of all target genes in at least one tissue. The relative abundance of *GA2ox* transcripts was compared in different tissues and a positive correlation was found between abundance and silencing (Fig. 4). Genes more efficiently silenced within a tissue were those most highly expressed.

### General characterization of transgenic plants

Transgenic plants from lines L1 and L5 were characterized for a number of vegetative and reproductive traits which were known to be influenced by GAs, such as germination, hypocotyl and stem elongation, and flowering. None of these parameters showed significant differences between transgenic and WT plants, including plant height (see Supplementary Figs S2 and S3 and Supplementary Table S4 at *JXB* online). Differences found in the hypocotyl lengths and fruit weight of line L5 were not reproduced in other experiments. A smaller number of seeds per fruit was only detected in line L1.

### Transgenic plants show facultative parthenocarpy

**Fig. 5.**
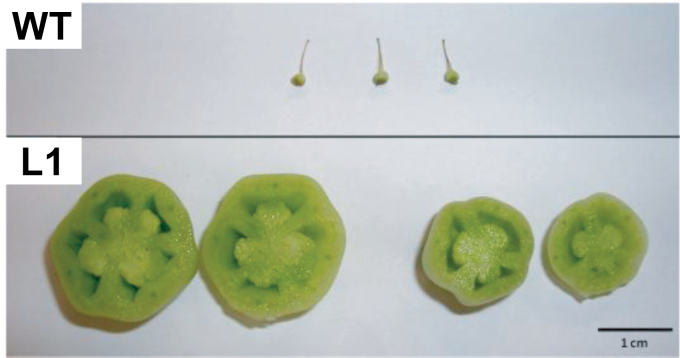
Photograph of 20-d-old unfertilized ovaries from WT and *35S::GA2ox/RNAi* plants (Line L1). (This figure is available in colour at *JXB* online.)

**Fig. 6.**
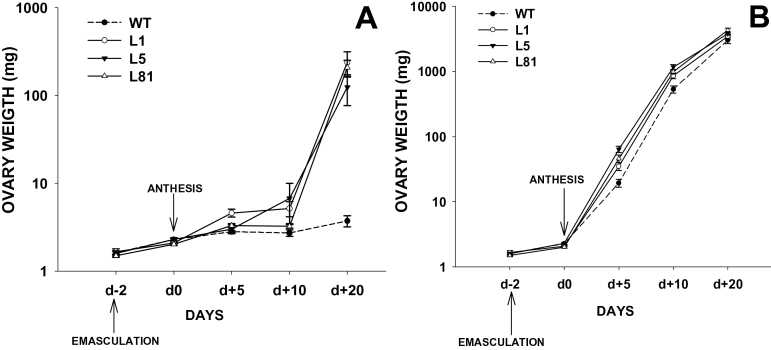
Development of fruits from emasculated or pollinated flowers in WT and *35S::GA2ox/RNAi* plants (Lines L1, L5, and L81). The staminal cones were removed two days before anthesis (d-2) to prevent self pollination (four trusses per plant, three flowers per truss). Some of them were hand-pollinated 2 d after emasculation (d0) and others were left unpollinated. Ovaries were collected on days –2, 0, 5, 10, and 20. Values are the mean of at least 15 ovaries per stage ±SE. (A) Non pollinated ovaries. (B) pollinated ovaries.

GAs are important for fruit-set and development (Seymour *et al.*, 2013). Usually, unfertilized tomato ovaries do not grow and develop into fruits although GA application can induce parthenocarpic development (Serrani *et al.*, 2007a). To determine if ovaries from transgenic lines had the ability to grow in the absence of fertilization, flowers were emasculated 2 d before anthesis, to prevent self-pollination, and the ovaries were collected after 20 d. While, in WT plants, the unpollinated ovaries did not grow, in transgenic plants a certain number of non-pollinated ovaries (from 5% to 37%, depending on the line and experiment) developed into parthenocarpic fruits (Table 1; Fig. 5). The development of hand-pollinated and emasculated ovaries was also compared for 20 d in WT and transgenic lines (Fig. 6). The development of emasculated ovaries was very different between transgenic and WT plants (Fig. 6A). After anthesis, the weight of WT emasculated ovaries did not change significantly while, in transgenic plants, ovary weight increased dramatically (more than 30 times) although they did not reached the weight of pollinated ovaries which were 10 times bigger. Pollinated ovaries developed in a similar way in transgenic and WT plants although ovaries from transgenic lines grew faster than WT ovaries before day 20, at which time the differences disappeared (Fig. 6B).

**Table 1.** Parthenocarpic capacity of wild type (WT) and transgenic *35S::GA2ox/RNAi* plants Parthenocarpic capacity was determined, in two separate experiments, by emasculating flowers 2 d before anthesis to prevent self-pollination (four trusses per plant, three flowers per truss) and collecting ovaries 20 d after anthesis. Parthenocarpic fruits were those bigger than 0.1g. Values in parentheses are the number of parthenocarpic fruits/total of emasculated ovaries. Weight data are the average of parthenocarpic fruits ±SE at 20 d after anthesis.

	Line	% Fruit-set	Parthenocarpic fruit weight d20 (g)
Experiment I	WT	0 (0/69)	–
	L1	37 (11/30)	0.62±0.1
	L5	15 (7/47)	0.81±0.15
	L81	34 (22/64)	0.59±0.13
Experiment II	WT	0 (0/101)	–
	L1	5 (4/79)	0.52±0.2
	L5	34 (35/102)	0.70±0.05

### *GA2ox* genes expression in pollinated and non-pollinated ovaries

**Fig. 7.**
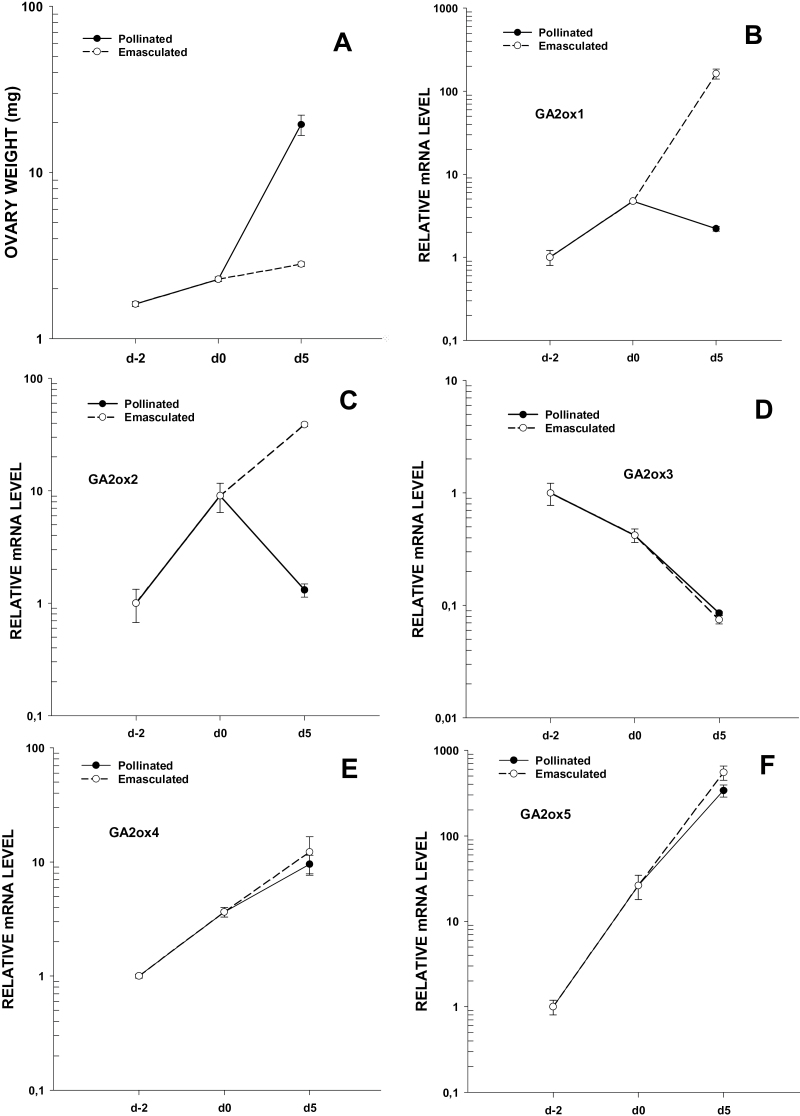
Expression of *GA2ox* genes in hand-pollinated and unpollinated ovaries in WT plants. The *SlActin* gene was used as an internal control for each tissue and values are means of three biological replicates ±SE. Results are expressed relative to the transcript level, at d-2 stage (2 d before anthesis), set as 1.0. (A) Weight of ovaries from pollinated and emasculated flowers used in this experiment; (B) *GA2ox1*; (C) *GA2ox2*; (D) *GA2ox3*; (E) *GA2ox4*; (F) *GA2ox5*.

The parthenocarpic fruit development found in transgenic plants suggests a role for GA 2-oxidases in the control of ovary growth. Therefore, the expression of the five *GA2ox* genes was compared between fertilized and unfertilized ovaries. Transcript levels of *GA2ox* genes were measured in hand-pollinated and emasculated ovaries from 2 d before anthesis until 5 d later when fruit-set had already been established, as shown in Fig. 7A. Pollinated ovaries after 5 d of anthesis are 10-times bigger than non-pollinated ones. Two of the genes (*GA2ox1* and *-2*) are differentially expressed in fertilized and non-fertilized ovaries after anthesis (Fig. 7B, C). The expression of *GA2ox1* and *GA2ox2* decreased after pollination but increased significantly in non-pollinated ovaries by up to 30 times. The other three genes *GA2ox3,-4*, and*-5* did not display differences in the expression in fertilized and non-fertilized ovaries (Fig. 7D–F). After anthesis, transcript levels of *GA2ox3* decreased while *GA2ox4* and *GA2ox5* increased (Fig. 7D–F), although no significant difference was found between fertilized and unfertilized ovaries. The patterns of expression suggest that *GA2ox1* and *GA2ox2* could play a role in fruit development as negative regulators of ovary growth.

### Transgenic plants show inhibition of branching

**Fig. 8.**
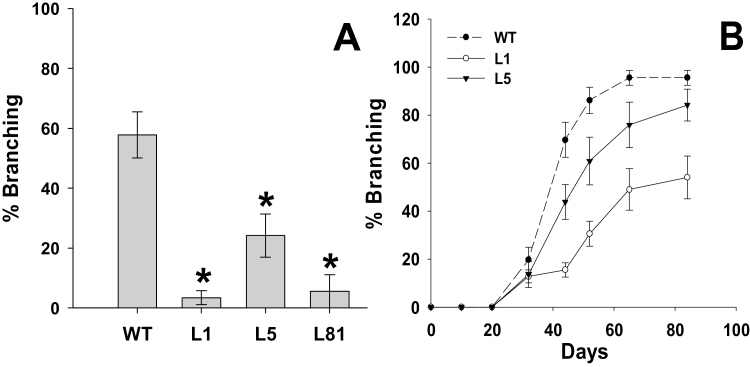
Branching pattern of WT plants and *35S::GA2ox/RNAi* plants. (A) % Branching of 90-d-old WT and *35S::GA2ox/RNAi* plants (Lines L1, L5, and L81). (B) % Branching of WT plants and *35S::GA2ox/RNAi* plants (Lines L1 and L5) for 85 d. % Branching values are represented as Mean of the percentage of lateral branches bigger than 0.5cm ±SE per plant. *, Significantly different from the WT plants (*P* <0.05).

As mentioned before, the main aspects of vegetative development of transgenic lines were similar to WT plants except for the branching pattern (see Supplementary Fig. S3 and Supplementary Table S4 at *JXB* online). A clear reduction in the number of lateral branches was found in transgenic lines compared with WT plants (Fig. 8A). To study this phenotype in more detail, the development of axillary buds into branches was followed in transgenic lines L1 and L5 and in WT plants for 85 d (Fig. 8B). After 40 d from sowing, more than 60% of the axils in WT plants had lateral branches while in transgenic lines the percentage of lateral branches was approximately 45% for L5 and less than 20% for L1. Over time the differences from the WT were reduced, mainly in L5. By the end of the experiment (85 d), the percentage of branching in WT plants was about 95%, while in L5 it was 80% and in L1 it was 55%.

### Branching inhibition is mediated by GAs

**Fig. 9.**
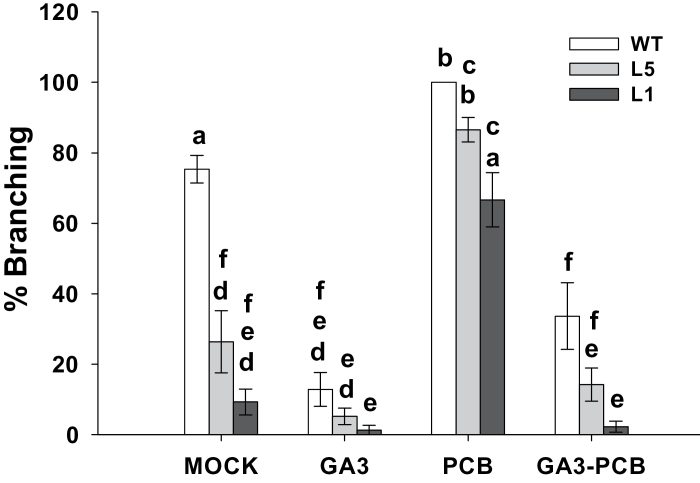
Branching pattern of WT plants and *35S::GA2ox/RNAi* plants (Lines L1 and L5) in response to GA content modifications. Adult plants (27-d-old) were treated with GA_3_ (10^–5^ M), PCB (10^–6^ M), and GA_3_+PCB for 16 d. % Branching values are represented as Mean of the percentage of lateral branches bigger than 0.5cm ±SE per plant. The same letter means there are no significant differences (*P* <0.05).

**Fig. 10.**
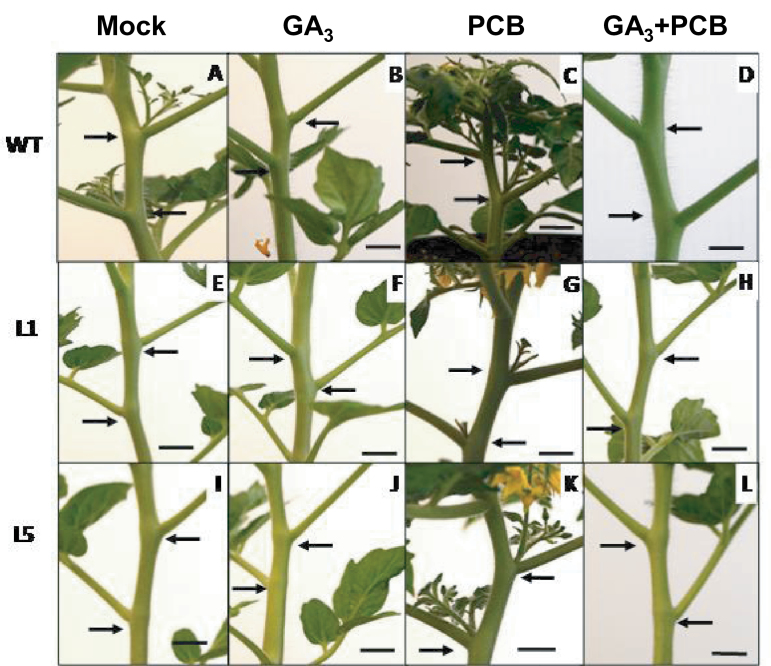
Photographs of the 3rd and the 4th axils of WT plants and *35S::GA2ox/RNAi* plants (Lines L1 and L5) in response to treatment with GA_3_ (10^–5^ M), PCB (10^–6^ M), and GA_3_+PCB. (A, E, I) WT, L1, and L5 untreated plants; (B, F, J) WT, L1, and L5 GA_3_-treated plants; (C, G, K) WT, L1, and L5 PCB-treated plants; (D, H, L) WT, L1, and L5 GA_3_+PCB- treated plants. Arrows point to axils 3 and 4 in each plant. Scale bars=1cm. (This figure is available in colour at *JXB* online.)

To test if branching inhibition was related to a modification of the GA content, GA levels were altered by the application of PCB (a GA biosynthesis inhibitor) and by GA_3_ application. In addition, plants were also treated simultaneously with GA_3_ and PCB to determine if the PCB action on branching was mediated by GAs. Figure 9 shows the percentage of branching of 52-d-old plants treated with a GA_3_, PCB or GA_3_+PCB solution for 16 d. As seen before, untreated transgenic plants (L1 and L5) had significantly fewer branches than WT, especially L1. Interestingly this phenotype was suppressed by PCB treatment in both transgenic lines while GA_3_ application was able to inhibit branching in the WT (Figs 9, 10). Moreover, branching stimulation induced by PCB treatment was completely suppressed by simultaneous GA_3_ application, indicating that the PCB effect on branching was due to a reduction in GA level and not to interaction with other pathways (Figs 9, 10).

### GA content in transgenic plants

GA 2-oxidases catalyse the deactivation of active GAs and their precursor. In order to evaluate the effect of GA2ox silencing on GA metabolism, GAs levels were quantified in ovaries and vegetative tissues. Fertilized ovaries were compared because they develop into fruits in both transgenic and WT plants. On the other hand, unfertilized ovaries of transgenic plants develop into parthenocarpic fruits while ovaries do not grow in the WT plants (Fig. 5). A higher content of active GA_4_ was found in the ovaries of transgenic plants (more than 3-fold in L1 and 2-fold in L5) and a 60% decrease was found in the levels of inactive GA_34_ compared with WT plants (Table 2). The active GA_1_ content was not altered in any of the transgenic lines although GA_20_ was significantly higher and GA_8_ significantly lower in L1.

**Table 2.** Gibberellin concentration (ng g^–1^ FW) in reproductive tissues (10-d-old pollinated ovaries) of WT and transgenic *35S::GA2ox/RNAi* plants (Lines L1 and L5) Results are the mean of five biological replicates±SE. *, Significantly different from WT (*P* <0.05).

Early 13-hydroxylation	WT	L1	L5
GA_19_	0.64±0.08	0.46±0.04	0.51±0.05
GA_20_	0.35±0.02	0.57±0.01*	0.42±0.03
GA_1_	0.30±0.02	0.29±0.03	0.27±0.02
GA_8_	1.65±0.03	1.20±0.05*	1.62±0.35
Non 13- hydroxylation			
GA_4_	0.36±0.02	1.21±0.09*	0.78±0.11*
GA_34_	0.032±0.001	0.013±0.001*	0.011±0.001*

No differences in GA levels were observed between transgenic and WT plants in apical shoots and stems in accordance with the absence of stem elongation differences (see Supplementary Fig. S3 and Supplementary Tables S4 and S5 at *JXB* online).

The branching phenotype found in transgenic plants and the branching stimulation induced by PCB treatment suggested that GA content could be enhanced in axillary buds of transgenic plants. The GA content of axillary buds in WT plants and L1, which showed the most extreme phenotype, was compared,. The level of active GA_4_ (but not GA_1_) increased about 2- fold in the axillary buds from axils 4 and 5 of transgenic plants (Table 3). In addition, the levels of inactive GA_29_ and GA_51_ were reduced to undetectable levels in transgenic plants, as expected from the reduced activity of the GA 2-oxidases.

**Table 3.** Gibberellin concentration (ng g^–1^ FW in axillary buds of WT and transgenic *35S::GA2ox/RNAi* plants (Line L1) Results are the mean of three biological replicates ±SE. *, Significantly different from the wild type (*P* <0.05). a: below detection level; n.r: no result due to weak signals or interfering ions. GA_34_ could not be quantified due to the lack of internal standard.

	3rd Axil		4th Axil		5th Axil	
	WT	L1	WT	L1	WT	L1
GA_53_	4.43±1.22	4.29±0.17	7.89±0.66	6.25±0.48	7.08±0.67	6.11±0.25
GA_44_	1.78±0.39	1.07±0.09	2.54±0.09	1.77±0.26*	2.38±0.08	2.17±0.17
GA_19_	2.06±0.44	0.74±0.03*	2.54±0.15	1.24±0.10*	3.70±0.20	2.78±0.44
GA_20_	0.026±0.005	0.020±0.002	0.05±0.008	0.02±0.007	0.05±0.02	0.03±0.002
GA_1_	0.059±0.006	0.069±0.003	0.090±0.013	0.081±0.014	0.15±0.03	0.15±0.02
GA_29_	0.026±0.015	a	0.004±0.003	a	0.013±0.003	a
GA_8_	n.r	n.r	n.r	n.r	n.r	n.r
GA_15_	0.97±0.24	0.58±0.06	1.03±0.30	0.98±0.16	1.03±0.28	0.27±0.20
GA_24_	0.133±0.035	0.058±0.011	0.12±0.03	0.10±0.01	0.06±0.01	0.05±0.03
GA_9_	0.129±0.013	0.121±0.012	0.17±0.06	0.14±0.014	0.31±0.03	0.17±0.02*
GA_4_	0.040±0.007	0.065±0.010	0.083±0.011	0.178±0.020*	0.07±0.01	0.20±0.04*
GA_51_	0.005±0.002	0.016±0.008	0.007±0.004	a	0.002±0.002	a

## Discussion

### Efficiency of gene silencing

The levels of active GAs are regulated by biosynthetic and catabolic enzymes like the GA 2-oxidases (GA2oxs) which, in tomato (*Solanum lycopersicum* L.), are encoded by a small multigenic family of five members with some degree of redundancy (Serrani *et al.*, 2007a). In order to investigate the roles of GA2ox enzymes in tomato development, the silencing of all five *GA2ox* genes was induced using a chimeric short-hairpin construct. The *35S::GA2ox/*RNAi transgene reduced GA2ox mRNA levels in tomato plants, although large differences in the efficiency of gene silencing were found (Figs 2, 3). The efficiency of gene silencing has generally been associated with the degree of identity between target and trigger RNA sequences (Katoch and Thakur, 2013). However, in this case, the differences in silencing do not correlate with the degree of sequence identity. On the other hand, a direct relationship between silencing and target abundance was found as previously described in other works (Miki *et al.*, 2005; Gallego-Giraldo, 2008; Gil-Humanes *et al.*, 2010; Shigemitsu *et al.*, 2012). Within a tissue, the silenced genes were always among the most abundantly expressed (Fig. 4). However, a gene that is expressed with a similar abundance in two different tissues may be silenced in only one of them. This discrepancy might be related to differences in the cellular components of the silencing machinery or to differences in the expression of the trangene between tissues.

### Effect of *GA2ox* silencing in GA metabolism

GA 2-oxidase enzymes catalyse the addition of a hydroxyl group in the C-2 carbon of active GA_1_ and GA_4_ and their immediate precursors, GA_20_ and GA_9_, respectively (Fig. 1), causing the loss of biological activity. Silencing of *GA2ox* genes is expected to produce an accumulation of active GAs and a decrease in the inactive products (GA_29_, GA_8_, GA_51_, and GA_34_). GA levels were quantified in four different tissues but in only two of them (ovaries and axillary buds) could significant alterations of GA metabolites be found. In the apical shoots, where none of the *GA2ox* genes were silenced, there were no changes in GA levels (see Supplementary Table S5 at *JXB* online). In stems, where only the expression of *GA2ox3* and *GA2ox5* was reduced, no differences in the GA content were detected (see Supplementary Table S5 at *JXB* online). This fact may be due to functional redundancy. In ovaries and axillary buds, as expected from a deficiency in GA 2-oxidases, significant decreases in inactive products were detected (Tables 2, 3) in both GA pathways (Fig. 1). There were, however, some exceptions, for instance, GA_8_ in ovaries of L5 did not show any significant reduction. The inactivation of GAs by GA 2-oxidases may include a second oxidation step (Thomas *et al.*, 1999) producing GA catabolites (GA-cat; Fig. 1). Since these GA catabolites could not be quantified and their levels also depend on the activity of GA 2-oxidases, the similarities in inactive GAs do not preclude differences in GA 2-oxidase activity. With respect to active GAs, while GA_4_ levels increased in both tissues of transgenic plants, GA_1_ levels remained unaltered, which was unexpected since both GA_4_ and GA_1_ are substrates of GA 2-oxidases. This may be due to the complexity of the pathway (Fig. 1). The suppression of GA 2-oxidases should increase the levels of GA_4_ and GA_1_ as well as their precursors GA_9_ and GA_20_ which are also substrates of the biosynthetic GA3ox enzyme. Due to the expected increase in these substrates, GA_9_ and GA_20_, the ratio of substrate/GA3ox may change, making GA3ox activity a limiting factor for this reaction in transgenic plants. In this context, our results could be explained by assuming that GA3ox enzymes have more affinity for the GA_9_ substrate, thus favouring the conversion of GA_9_ to GA_4_ instead of converting GA_20_ to GA_1_.

### Phenotypes of transgenic plants

The alteration of active GA levels is known to have an important impact on plant height. Tomato plants with an enhanced content of active GAs, like GA20ox over-expressing plants, show a significant increase in plant height (Garcia- Hurtado *et al.*, 2012). Our transgenic *35S::GA2ox/*RNAi plants did not show significant alterations in plant stature (see Supplementary Fig. S3 and Supplementary Table S4 at *JXB* online). This is consistent with the poor silencing of *GA2ox* genes (Fig. 2) and unmodified GA levels (see Supplementary Table S5 at *JXB* online) in apical shoots and stems. However, our transgenic plants showed two interesting phenotypes: facultative parthenocarpic capacity and inhibition of branching.

### *GA2ox* silencing induces parthenocarpic fruit growth in transgenic plants

Fruit-set in tomato depends on GA biosynthesis and signalling (de Jong *et al.*, 2009). Previous work by Garcia- Hurtado *et al.* (2012) showed that enhancing the GA content in the ovary of GA20ox over-expressing tomato plants induce facultative parthenocarpy. A similar phenotype was also found in a quintuple loss-of-function mutant for *GA2ox* genes in *Arabidopsis thaliana* (Rieu *et al.*, 2008). In our *35S::GA2ox*/RNAi transgenic plants a proportion of unfertilized ovaries (between 5% and 37%) developed into parthenocarpic fruits (Fig. 5; Table 1) while ovaries from WT plants did not grow. This phenotype correlates with the silencing of the *GA2ox* family (Fig. 3) and the increase in the concentration of active GA_4_ (Table 2) in the ovaries. This enhancement in GA_4_ content was found to be at least twice the WT content. This is particularly significant since it takes place in fertilized ovaries where GA content is already high and suggests that GA2oxs are important in the regulation of GA levels in the ovary of tomato.

The evolution of ovary growth in WT and transgenic plants has also been compared. Unfertilized ovaries from transgenic plants grew faster and bigger (30 times more) than WT ovaries and the differences between them became apparent 5 d after anthesis (Fig 6A). Hand-pollinated ovaries from transgenic plants also developed more rapidly than WT plants from day five to day 20 after anthesis. Beyond this stage, any differences between transgenic and non-transgenic ovaries disappeared (Fig. 6B). This suggests that the increase in active GA_4_ content, mediated by *GA2ox* silencing, has an impact in the early stages of fruit development.

### *GA2ox1* and *GA2ox2* genes are differentially expressed in pollinated and non-pollinated ovaries

Fruit-set can be defined as the developmental switch that turns an ovary into a growing fruit and it depends on the fertilization of ovules. In the absence of fertilization, a decrease in active GA levels has previously been described (Mariotti *et al.*, 2011) and the development of the ovary into a fruit is consequently prevented. However, it was found that the silencing of the *GA2ox* genes induces the growth of unfertilized ovaries, suggesting that these genes may be involved in preventing the growth of WT unfertilized ovaries. When the expression pattern of *GA2ox* genes was compared between pollinated and non-pollinated ovaries it was noted that only two of them, *GA2ox1* and *GA2ox2,* showed significant differences between both developmental pathways. These two genes were significantly up-regulated in emasculated ovaries at day 5 and down-regulated in pollinated ones (Fig. 7B, C) showing an inverse correlation with the growth of the ovary (Fig. 7A). According to our results, it appears that *GA2ox1* and *GA2ox2* genes may contribute to the reduction of GA levels taking place in unfertilized ovaries preventing their growth. On the other hand, fertilization may trigger the growth of the ovary partly by repressing *GA2ox1* and *GA2ox2.* Similar differences in the expression pattern of some *GA2ox* genes in fertilized and unfertilized ovaries have also been seen in *Arabidopsis thaliana* (Dorcey *et al.*, 2009) as well as in pea (Ozga *et al.*, 2009). By contrast, this difference in expression was not detected by Serrani *et al.* (2007a) in tomato ovaries. This discrepancy may be due to differences in their sampling and the method used to analyse gene expression (semi-quantitative RT-PCR).

However, no differences of expression were found for *GA2ox3, -4*, and *-5* genes in pollinated and emasculated ovaries (Fig. 7D–F), suggesting that they are not controlling ovary growth. Their specific roles in the tomato ovary remain unknown.

### *GA2ox* silencing inhibits lateral branching in transgenic plants

Despite the fact that transgenic *35S::GA2ox/RNAi* plants were not modified in stem length or GA levels in apical shoots and stems (see Supplementary Fig. S3 and Supplementary Tables S4 and S5 at *JXB* online), they showed a significant inhibition of axillary bud outgrowth (Fig. 8A, B). This branching inhibition was reversed by inhibiting GA biosynthesis (Figs 9, 10C, G, K). The role of GAs in branching was also supported by the inhibitory effect of GA_3_ application on WT plants (Figs 9, 10B, F, J). These findings suggest that this phenotype might be mediated by an increase in the axillary bud GA content. Indeed, a significant increase in the amount of bioactive GA_4_ in the axillary buds (Table 3) of transgenic line L1 was confirmed, indicating that the phenotype of transgenic plants was due to an alteration in the active GA content. Moreover, a reduction of inactive GAs (GA_29_ and GA_51_) to undetectable levels was found in the transgenic plants, as expected from *GA2ox* silencing (Table 3). These results suggest that branching inhibition is mediated by GAs and that GA2ox enzymes are important in regulating the levels of active GAs in the axillary buds of tomato.

Despite the fact that branching inhibition was not mentioned in a quintuple loss-of-function mutant for *GA2ox* genes in *Arabidopsis thaliana* (Rieu *et al.*, 2008) there are hints in support of the inhibitory role of GA in branching in tomato and other plant species. In *procera,* a GA constitutive response tomato mutant, the oldest axillary buds are repressed (Bassel *et al.*, 2008). *Pelargonium* transgenic plants that over-express a DELLA gene from rose displayed a significant reduction of plant height and also a significant induction of branching (Hamama *et al.*, 2012). In other species such as citrus and pea, GA-defective mutants produce more lateral branches than WT plants (Fagoaga *et al.*, 2007; de Saint Germain *et al.*, 2013). On the other hand, dwarf transgenic plants that over-express the catabolic enzymes GA2ox, in rice (Lo *et al.*, 2008), *Paspalum notatum* Flugge (Agharkar *et al.*, 2007), and aspen (Mauriat *et al.*, 2011), have also been found to produce more lateral branches than WT plants.

Recently, Rameau *et al.* (2015) proposed a new model for hormonal regulation of branching which includes GAs, together with the already well-known regulators of branching auxin, strigolactones (SLs), cytokinins (CKs), and BRC1. Our results, together with previous works, support the inclusion of GAs in this model as negative regulators of branching. Despite all the evidence, the inhibition of shoot branching by GAs is more commonly described as a ‘side-effect phenotype’ instead of a ‘classical GA phenotype’ and, therefore, little information is available about the interaction of GAs with auxins, SL, CKs, and BRC1 with regard to this phenotype in particular, and especially in tomato. Unlike other reports, the only vegetative phenotype observed in our plants is the inhibition of branching suggesting that the effect of GAs in branching is specific and localized and it is not due to the reallocation of resources in the plant.

Although the role of GAs in branching is clear, to determine how the GAs inhibit the outgrowth of lateral buds remains an important challenge: Is the GAs action mediated by its interaction with the other regulators of the branching model or not? Although at this point the possibility cannot be ruled out of GAs acting through an independent pathway, there is evidence supporting the GA interaction with other branching model components (auxins, SL, CKs). A positive interaction of GA with auxin transport has been found in *Arabidopsis thaliana* (Willige *et al.*, 2011) and aspen plants (Mauriat *et al.*, 2011). In our transgenic tomato plants, the GA content was not significantly altered in the stem, although more work is needed in order to determine conclusively whether the auxin gradient remains unaltered. The interaction between GAs and SLs, has previously been reported in rice (Nakamura *et al.*, 2013) although, in pea, de Saint Germain *et al.* (2013) suggest that GAs and SLs act independently to regulate branching. Moreover, in tomato, GAs and cytokinins have a mutual antagonist interaction in the regulation of processes such as the complexity and serration of leaves according to Fleishon *et al.* (2011), but this antagonism has not yet been studied in relation to branching.

Given the complex interactions between the hormones involved in branching found in other species such as pea (Young *et al.*, 2014), further work will be necessary to clarify the position of GAs within the branching model of tomato. Currently, our efforts are focused on the generation of genetic resources, such as relevant hormonal tomato mutants and the implementation of biochemical and physiological tools, with the intention of testing some of these hypotheses in the future.

In summary, this work demonstrates that GA 2-oxidases regulate gibberellin levels in the ovaries and axillary buds of tomato plants and their silencing is responsible for parthenocarpic fruit growth and branching inhibition.

## Supplementary data

Supplementary data can be found at *JXB* online.

Supplementary Table S1. Sequences of chimeric fragment used for silencing *GA2ox* genes.

Supplementary Table S2. Primers used for the generation of constructs.

Supplementary Table S3. Primers used for RT-qPCR reactions.

Supplementary Table S4. Vegetative and reproductive phenotypes of transgenic *35S::GA2ox*/RNAi plants (Lines L1 and L5).

Supplementary Table S5. Gibberellin concentration (ng g^–1^ FW) in the apical portion of plants and stems of wild-type plants (WT) and transgenic *35S::GA2ox*/RNAi plants (Line L1).

Supplementary Fig. S1. Schematic representation of shRNA2ox fragment construction.

Supplementary Fig. S2. Germination characteristics of WT and transgenic *35S::GA2ox/RNAi* plants (Lines L1 and L5).

Supplementary Fig. S3. Photograph of representative adult (52-d-old) WT and *35S::GA2ox/RNAi* plants (Lines L1 and L5).

Supplementary Data

## Abbreviations:

GA: gibberellin
GA2ox: gibberellin 2-oxidase
GA3ox: gibberellin 3-oxidase
GA13ox: gibberellin 13-oxidase
GA20ox: gibberellin 20-oxidase
PCB: paclobutrazol
RT–qPCR: reverse transcription-quantitative PCR
SE: standard error
shRNA: short-hairpin RNA
WT: wild type.
