# Supplementary material for: Silencing C19-GA 2-oxidases induces parthenocarpic development and inhibits lateral branching in tomato plants
Source: J Exp Bot. 2015 Jun 19;66(19):5897–910. doi: 10.1093/jxb/erv300 (PMC4566981; doi:10.1093/jxb/erv300)
Supplement: Supplementary Data [file supp_66_19_5897__index.html]

Silencing C19-GA 2-oxidases induces parthenocarpic development and inhibits lateral branching in tomato plants — Silencing C19-GA 2-oxidases induces parthenocarpic development and inhibits lateral branching in tomato plants — Silencing C19-GA 2-oxidases induces parthenocarpic development and inhibits lateral branching in tomato plants — Supplementary Data 

# Silencing C19-GA 2-oxidases induces parthenocarpic development and inhibits lateral branching in tomato plants

## Supplementary Data

Data files

- Supplementary Data - Supplementary Data
