# Supplementary material for: Silencing C19-GA 2-oxidases induces parthenocarpic development and inhibits lateral branching in tomato plants
Source: J Exp Bot. 2015 Jun 19;66(19):5897–910. doi: 10.1093/jxb/erv300 (PMC4566981; doi:10.1093/jxb/erv300)

Journal of Experimental Botany

"Silencing C19-GA 2-oxidases Induces Parthenocarpic Development and Inhibits Lateral Branching in Tomato Plants".

Liliam Martínez-Bello; Thomas Moritz; Isabel López-Díaz

Supplementary Data

Table S1. Sequences of chimeric fragment used for silencing *GA2ox* genes

Gene	Fragment length (pb)	Fragment sequence (5'-3')
GA2ox1	188	AATTGGATTTGGAGAGCATACAGACCCTCAGATCTTG ACCATCTTAAGATCAAACGATGCTCCTGGCCTACAAAT ATCAACTCAACAAGGATTGTGGGTCCCCGTATCACCTC ACCCGAATACTGCTTTTTCCATCTTCGTTGGAGACACT TTACAGGCTCTGACAAATGGAAGGTTTAAGAGTGTGA
GA2ox3	123	GACATAGAGTGGTGTCAATAAATTCATGGAAAAGTAG AATGTCCATGATGTACTTTGCAGCTCCTGCTCTTGGCG CTTGGATAAGTGCTCCTCCTCAAATCAACAATATTACT AATATTTATA
GA2ox4	163	ACCTCATCGTTAATGCCTGCGAAGAATTCGGATTCTTC AAAGTCATAAACCATGATGTTCCTATGGAATTCATAA GTAAACTCGAATCCGAAGCCGTTAAATTCTTCTCCTCT CCCCTCTCTGAGAAACTAAAGGCAGGGCCTGCTGATC CTTTTGGTTATGG

Table S2. Primers used for generation of constructions

Primers	Sequence (5'-3')
GA2ox4-af	CTCGAGTCTAGAACCTCATCGTTA
GA2ox4-ar	TCCAAATCCAATTTCTTATTGCCA
GA2ox1-bf	TGGCAATAAGAAATTGGATTTGGA
GA2ox1-br	CCACTCTATGTCTCACACTCTTAA
GA2ox3-cf	TTAAGAGTGTGAGACATAGAGTGG
GA2ox3-cr	ATCGATGGTACCTATAAATATTAG
attB1shRNA2oxf	GGGGACAAGTTTGTACAAAAAAGCAGGCTCTCGAGTCTAGAACCTCA
attB2shRNA2oxr	GGGGACCACTTTGTACAAGAAAGCTGGGTATCGATGGTACCTATAAA

Table S3. Primers used for RT-qPCR reactions.

Primers	Sequence (5’-3’)
GA2ox1f	GGCATGTAAGATATTAGAATTGA
GA2ox1r	TTAATCCGTAGTAGAGAATCAGA
GA2ox2f	TCCCACCATGTTAGGAATTAT
GA2ox2r	TGTAAGCCCACAATGAGCATC
GA2ox3f	GACCCTTCTACTTTCAGCTC
GA2ox3r	AAATTGAATTGTCTTCTATCCA
GA2ox4f	ATGGAAGGAAAAGACAGTTTA
GA2ox4r	CTTTTCTCAAATAGGACCAAC
GA2ox5f	GATCACTTACCAATAATCAACAG
GA2ox5r	CGTCATGGTTTACGACTTTA
SlActinf	GAAACCTTCAACACCCCTGC
SlActinr	TGGGAACTGTGTGGCTGACA

Table S4. Vegetative and reproductive phenotypes of transgenic *35S::GA2ox*/RNAi plants (Lines L1 and L5).

Data is represented as Mean of at least 10 plants ± SE, except when otherwise stated.

VEGETATIVE PHENOTYPES			
**Parameter**	**WT**	**L1**	**L5**
Hypocotyl length (cm) [a]	1,57±0,04	1,62±0,07	1,36±0,04*
Root length (cm) [a]	5,90±0,61	6,14±0,61	4,88±0,42
Height to first inflorescence (cm)	11,86±0,47	12,70±0,59	10,95±0,61
Number of leaves to first inflorescence	7,82±0,42	7,70±0,35	7,18±0,30
Stem diameter (cm) [b]	0,65±0,03	0,62±0,01	0,65±0,02
Internode length (cm) [b]	1,88±0,11	1,92±0,13	1,86±0,10
Leaf size (cm^2) [c]	179,1±15,4	210,2 ± 10,4	183,8 ± 8,5
Number of leaflets (n) [c]	5,70±0,40	6,10±0,33	5,64±0,39
Fresh weight roots (g) [d]	6,52 ± 0,38	7,03 ± 0,85	7,16 ± 0,57
**REPRODUCTIVE PHENOTYPES**			
**Parameter**	**WT**	**L1**	**L5**
Number of flowers in the two first inflorescences	13,4 ± 0,3	12,8 ± 0,7	13,4 ± 0,5
Days to anthesis [e]	31,3 ± 1,2	32,0 ± 0,7	29,4 ± 0,5
Days to colour break [f]	81,63±0,84	83,60±0,79	82,60±0,77
Number of fruits per plant [g]	46,05±2,40	43,00±2,95	53,11±3,49
Number of seeds per fruit [g]	14,50±0,67	10,66±0,58*	14,41±0,67
Fruit weight (g) [g]	2,98±0,07	2,83±0,07	2,60±0,07*
Number of locules per fruit [g]	2,81±0,02	2,88±0,03	2,88±0,03

a: Means ± SE of hypocotyl and root length of 7 day-old seedlings are represented.

WT (N=16); L1 (N=12); L5 (N= 13).

b: The stem diameter and internode length correspond to that of the fifth internode.

c: The leaf size and number of leaflets are those from the fifth leaf.

d: Roots from 90 day-old plants (9 plants per line).

e: Number of days required for the first flower to open.

f: Number of days required for the first fruit to reach maturity.

g: These data correspond to all mature fruits collected along 2 months.

Table S5. Gibberellin concentration (ng x g FW^{-1}) in the apical portion of plants and stems of wild-type plants (WT) and transgenic *35S::GA2ox*/RNAi plants (Line L1).

Results are mean of biological replicates ± SE.

*, significantly different from wild type (P<0.05)

	Apical shoots			Stem	
**Early 13-hydroxylation**	**WT**	**L1**		**WT**	**L1**
**GA_{53}**	-	-		2.66 ± 1.21	2.02 ± 0.25
**GA_{44}**	-	-		1.99 ± 0.38	1.58 ± 0.16
**GA_{19}**	0.08 ± 0.07	0.08 ± 0.06		1.74 ± 0.03	0.98 ± 0.12
**GA_{20}**	0.038 ± 0.03	0.05 ± 0.02		0.05 ± 0.003	0.05 ± 0.007
**GA_{1}**	0.06 ± 0.03	0.03 ± 0.01		0.52 ± 0.02	0.56 ± 0.04
**GA_{8}**	0.54 ± 0.17	0.58 ± 0.20		1.48 ± 0.01	1.33 ± 0.02
**GA_{29}**	-	-		2.18 ± 1.27	0.58 ± 0.0
**Non 13-hydroxylation**					
**GA_{12}**	-	-		-	-
**GA_{15}**	-	-		5.94 ± 1.08	6.30 ± 0.32
**GA_{24}**	-	-		0.32 ± 0.02	0.24 ± 0.01
**GA_{9}**	-	-		0.07 ± 0.04	0.04 ± 0.0
**GA_{4}**	0.06 ± 0.05	0.09 ± 0.05		2.27 ± 0.07	2.39 ± 0.03
**GA_{34}**	0.005 ± 0.002	0.013 ± 0.01		-	-
**GA_{51}**	-	-		2.40 ± 0.13	2.42 ± 0.09

Figure S1. Schematic representation of shRNA2ox fragment construction

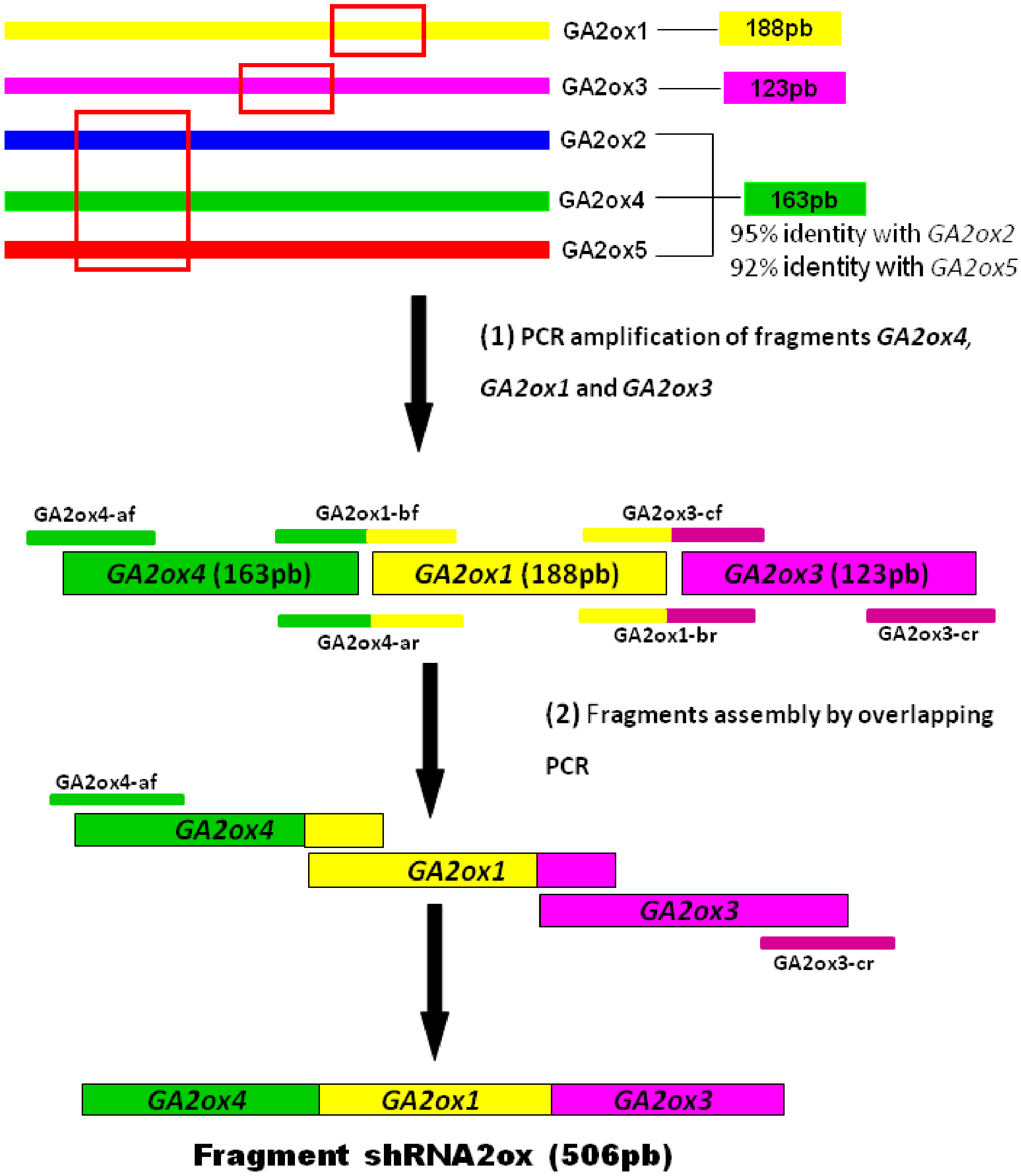

Figure S2. Germination characteristics of WT and transgenic *35S::GA2ox/RNAi* plants (Lines L1 and L5).

A total of 180 seeds per line (from three different batches) were analyzed for germination during 96 hours. The percentage values represented are the average of the three independent batches ± SE

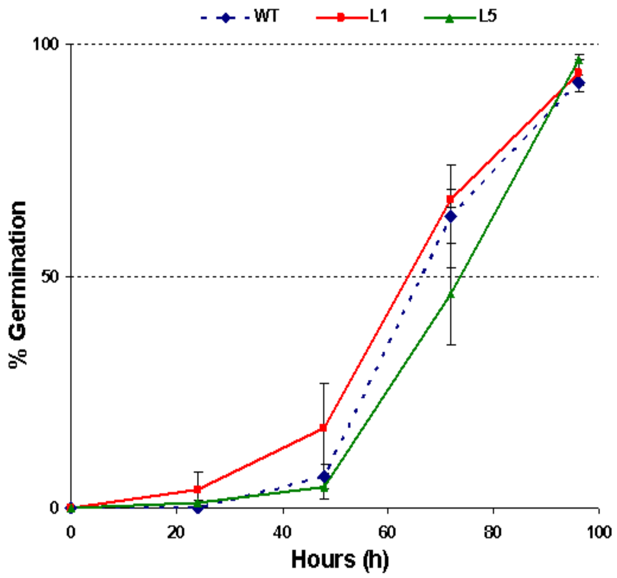

Figure S3. Photograph of representative adult (52 day-old) WT and *35S::GA2ox/RNAi* plants (Lines L1 and L5).

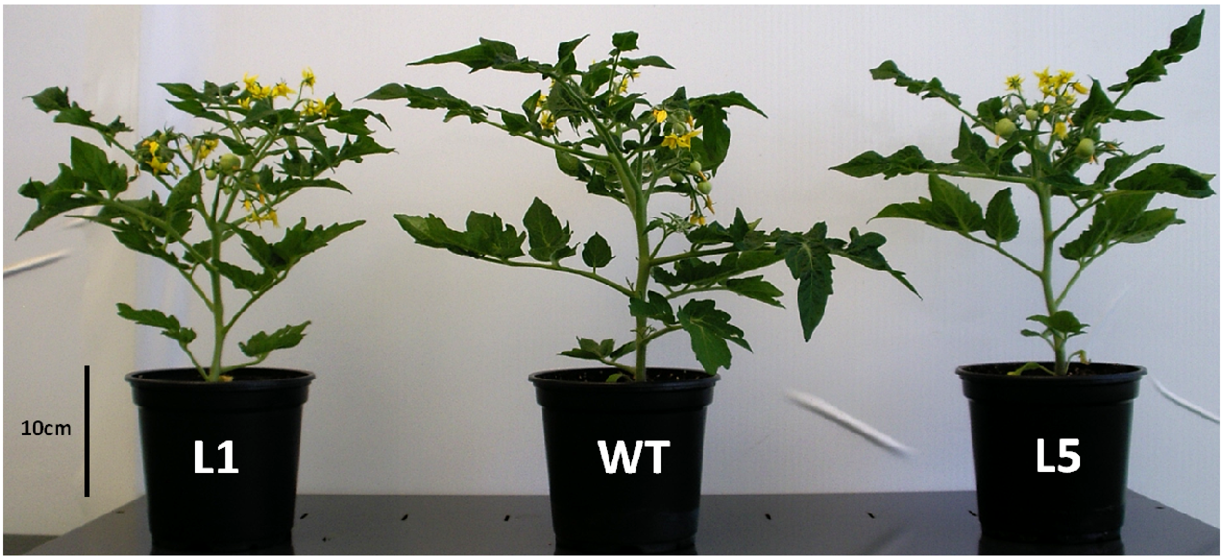

Supplement: Supplementary Data [file supp_erv300_jexbot131466_file001.pdf]
